# Supplementary material for: Sex hormone profiles and associated factors among adult tuberculosis patients at Gondar town, northwest Ethiopia: A comparative cross-sectional study
Source: PLoS One. 2026 Feb 6;21(2):e0340631. doi: 10.1371/journal.pone.0340631 (PMC12880706; doi:10.1371/journal.pone.0340631)
Supplement: S1 Table — (DOCX) [file pone.0340631.s001.docx]

**Questionnaire (English version)**

First, I would like to acknowledge you for your time and support to help me fill this questionnaire. The aim of this questionnaire is to gather information for the study to assess and compare sex hormone profile among adult Tuberculosis patients and apparently healthy controls and identify associated factor. The success of this research relies on you by providing timely and right information upon request. Finally, you are kindly requested to provide information honestly and responsibly. Therefore, are you voluntary to respond these questions?

Yes______ Signature______________

Please encircle the answer

| Questionnaire  Study code _____________________  Date of visit: __ __/__ ___/__ __ __ __ GC (day/month/year)  Name of institution __________________________________ | | |
| --- | --- | --- |
| No. | | Variable |
| 1, | | Demographical data |
| 1.1 | | Sex: 1) Male 2) Female |
| 1.2 | | Age: __________________ |
| 1.3 | | Weight: ______________ Kg Height: ___________ cm |
| 1.4 | | Marital status: 1) single 2) married 3) widowed 4) divorced |
| 1.5 | | Education status: 1) unable to read and write 2) primary school (grade1-8)  3) secondary school (grade 9-12) 4) higher education (college and above) |
| 1.6 | | Residency: 1) rural 2) urban |
| 1.7 | | Occupation: 1) House wife 2) Governmental employed 3) Self-employed 4) Student 5) unemployed 6) other (specify_________) |
| 1.8 | | Income (Ethiopian Birr): _______________ |
| 2. | | Behavioral activities |
| 2.1 | | Do you drink alcohol? 1) No 2) Yes 3) stopped |
| 2.2 | | Do you smoke cigarette? 1) No 2) Yes 3) stopped |
| 2.3 | | If you answered yes to question 2.2, for how many years __________? |
| 2.4 | | If you answered yes to question 2.2, how many cigarettes do you smoke per day______? |
| 2.5 | | Do you have physical exercise? 1) No 2) Yes  If yes ___times per week? for ___ minutes |
| 2.6 | | How often do you include healthy foods in your diet? 1) No 2) sometimes 3 most of time/ usually 4) always  (A healthy diet includes: wheat, barley, rice, grains, fruits, vegetables, meat, fish, eggs, milk and a spoonful of iodized salt are included in a typical daily diet). |
| 2.7 | | What is your usual food source? 1) Fruits or vegetables 2) Animal products 3) Both |
| 2.8 | | Is there a history of medical conditions in your family? 1) No 2) Yes  If yes, please specify: 1) Diabetes 2) Asthma 3) hypertensive  4) Other (please specify) ______ |
| 3 | | Sign and symptom assessment |
| 3.1 | Blood pressure? ______/_________(SBP/DBP) mmHg | |
| 3.2 | Menstrual irregularities (only for reproductive age women) | |
| 3.2.1 | How many days does your period last? _________________ (days) | |
| 3.2.2 | By how many days does your period come again (cycle)? ____________(days) | |
| 3.2.3 | How long has it been since your period? ___________ (days) | |

**Data collection sheet**

| A, Medical history extraction sheet for TB patients | |
| --- | --- |
| 1 | Type of TB classification? 1) Pulmonary extra pulmonary |
| 2 | \| Treatment status? 1) New 2) on treatment for _____months or ______days? \| \| --- \| \|  \| |
| 3 | Do you have taken drugs other than anti-TB drugs? 1) yes 2) no |
| 4 | Do you have history of TB? 1) Yes 2) no |
| 5 | Have you had medical illness? 1) No 2) yes  If yes type of illnesses? 1) Malaria 2) Giardiasis 3) other (specify_________) |
| 6 | Do you have vomit? 1) Yes 2) No |
| 7 | Do you have nausea? 1) yes 2) no |
| 8 | Loss of appetite? 1) yes 2) no |

| B: Laboratory test result assessment sheet | | | |
| --- | --- | --- | --- |
| No. | parameters | Value | Remark |
| 1 | Testosterone |  |  |
| 2 | Estradiol |  |  |
| 3 | Progesterone |  |  |
| 4 | LH |  |  |
| 5 | FSH |  |  |
| 6 | Cortisol |  |  |
