# Supplementary material for: Sex hormone profiles and associated factors among adult tuberculosis patients at Gondar town, northwest Ethiopia: A comparative cross-sectional study
Source: PLoS One. 2026 Feb 6;21(2):e0340631. doi: 10.1371/journal.pone.0340631 (PMC12880706; doi:10.1371/journal.pone.0340631)
Supplement: Attachment — Submitted filename: Response to Reviewers.docx [file pone.0340631.s005.docx]

October 1, 2025

**Sex hormone profiles and associated factors among adult tuberculosis patients at Gondar town, northwest Ethiopia: A comparative cross-sectional study**

**Manuscript ID: PONE-D-25-40268R1**

First of all, the authors would like to thank the PLOS ONE Academic Editorial Team, the editor who handled this manuscript, and the reviewers who have been reviewing, giving swift and valuable comments and suggestions to improve the quality of the manuscript. We have considered the given comments, and the corresponding revisions have been incorporated into the updated version of the manuscript. Your expertise and guidance have been pivotal in enhancing the overall quality of our manuscript. We have incorporated all comments and suggestions of the academic editor/reviewers. Lastly, we kindly invite to take a look the point-by-point response below.

**Point-by-point response for to editor’s/ reviewer’s comment**

**Point-by-point response for editor’s comment**

**Editor’s comment:** 1. Please ensure that your manuscript meets PLOS ONE's style requirements, including those for file naming.

**Authors’ response:** Dear editors. We thank the Academic Editor for this important reminder. We have carefully reviewed the PLOS ONE style requirements and updated our submission accordingly.

**Editor’s comment:** 2. In the online submission form, you indicated that [Most of data generated or analyzed during this study are included in this manuscript. Addition research data set associated with a paper is available, can be accessed up on request to the corresponding author.]. All PLOS journals now require all data underlying the findings described in their manuscript to be freely available to other researchers, either 1. In a public repository, 2. Within the manuscript itself, or 3. Uploaded as supplementary information.

**Authors’ response:** Dear editors. We thank the Editor for this clarification regarding PLOS ONE’s data availability requirements. In compliance with the journal’s policy, we confirm that most of the data underlying the findings of our study are fully included within the manuscript itself. We have revised the Data Availability Statement accordingly to accurately reflect this.

**Editor’s comment:** 3. PLOS requires an ORCID iD for the corresponding author in Editorial Manager on papers submitted after December 6th, 2016. Please ensure that you have an ORCID iD and that it is validated in Editorial Manager.

**Authors’ response:** Dear editors. We thank the Editor for this reminder. The corresponding author has created an ORCID iD, and it will be added and validated in Editorial Manager during the revised submission.

**Editor’s comment:** 4. Your ethics statement should only appear in the Methods section of your manuscript. If your ethics statement is written in any section besides the Methods, please move it to the Methods section and delete it from any other section. Please ensure that your ethics statement is included in your manuscript, as the ethics statement entered into the online submission form will not be published alongside your manuscript.

**Authors’ response:** Dear editors. We thank the Editor for this guidance. We have revised the manuscript accordingly our ethics statement now appears only in the Methods section and has been removed from all other sections.

**Editor’s comment:** 5. Please include a separate caption for each figure in your manuscript.

**Authors’ response:** Dear editors. We thank the Editor for pointing this out. We have revised the manuscript to include a clear and separate caption for each figure, following PLOS ONE’s formatting requirements.

**Editor’s comment:** 6. If the reviewer comments include a recommendation to cite specific previously published works, please review and evaluate these publications to determine whether they are relevant and should be cited. There is no requirement to cite these works unless the editor has indicated otherwise.

**Authors’ response:** Dear editors. We thank the Editor for this clarification. We have carefully reviewed the suggested references from the reviewers and evaluated their relevance to our study.

**Editor’s comment:** 7. Please review your reference list to ensure that it is complete and correct. If you have cited papers that have been retracted, please include the rationale for doing so in the manuscript text, or remove these references and replace them with relevant current references. Any changes to the reference list should be mentioned in the rebuttal letter that accompanies your revised manuscript. If you need to cite a retracted article, indicate the article’s retracted status in the References list and also include a citation and full reference for the retraction notice.

**Authors’ response:** Dear editors. We thank the Editor for this important reminder. We have carefully reviewed our reference list to ensure completeness and accuracy.

**Point-by-point response for Reviewer #1**

**Reviewer’s comments:**

Title:
**Reviewer’s comments:** What is 2024 in the title? This is not clear to the reader, suggested to remove it in the title. The reader will find out about the year the study was done within the document.

**Authors’ response:** Dear Reviewer #1, we thank the reviewer for this helpful observation. We agree that including “2024” in the title may cause confusion for readers, as the study period is already described in the manuscript. Accordingly, we have removed “2024” from the title to improve clarity.

**Abstract:**

**Reviewer’s comments:** Background of abstract: Is not clear and does not show the gap clearly. For example, the author written that the previous reports have conflicting results, so which results (need to be clear to the reader). The author should rewrite it and clearly show the gap.

**Authors’ response:** Dear Reviewer #1, We thank the reviewer for this important comment. We agree that the original background section of the abstract did not sufficiently specify the nature of the conflicting findings. To address this, we have revised the section to clearly describe the inconsistencies reported in previous studies and to better highlight the gap.

**Reviewer’s comments**: Methods of abstract: This study as specified is a comparative study and not a case control study, so advised not to use cases and control rather can say specifically as TB positive patients (or study group) and TB negative individual (comparative group). This should be changed throughout the document.

**Authors’ response:** Dear Reviewer #1, we sincerely thank the reviewer for this valuable comment. We agree with the suggestion and have revised the terminology throughout the abstract and the manuscript. Instead of referring to “cases” and “controls,” we now describe the groups as “TB positive patients (study group)” and “TB negative individuals (comparative group)” for clarity and accuracy.

**Reviewer’s comments**: Line 33: I hope there are more than five health institutions/hospital in Gondar town, so better say “selected five hospital/health institutions”

**Authors’ response:** Dear Reviewer #1, we thank the reviewer for this thoughtful observation. We agree with the suggestion and have revised the sentence at line 33 to read in five selected health institutions in Gondar town.

**Reviewer’s comments**: The author need to specify the participants were matched by which characteristics.

**Authors’ response:** Dear Reviewer #1, we thank the reviewer for this important comment. The participants in our study were matched by age, and this has been indicated in the revised manuscript.

**Reviewer’s comments**: Line 34 - 35: Specify in summery the kind of data that were collected

**Authors’ response:** Dear Reviewer #1. We thank the reviewer for this helpful suggestion. In the summary section, we have now specified the types of data collected, including sociodemographic, clinical, anthropometric, and behavioral characteristics.

**Reviewer’s comments:** Line 36: State in summary how hypogonadism was defined in this study

**Authors’ response:** Dear Reviewer #1. We thank the reviewer for this helpful suggestion. We have revised the abstract (line 36) to include a concise summary of how hypogonadism was defined in this study.

**Reviewer’s comments:** Line 37 – 38: State the statistical software used for data analysis ( ie. STATA version…., SPSS version etc)

**Authors’ response: Dear Reviewer #1**. We thank the reviewer for this comment. We have now specified in the abstract of manuscript that SPSS version 25.0 was used for statistical analysis.

**Reviewer’s comments:** Results of the abstract: Indicate p-value in brackets for all results presented (p = ….)

**Authors’ response: Dear Reviewer #1**. We thank the reviewer for this suggestion. We will indicate the p-values in brackets for all results presented in the abstract of revised manuscript.

**Reviewer’s comments:** Line 41: What do you mean by “New”. If meant newly diagnosed TB patients better write in full for the reader to be able to follow and understand

**Authors’ response: Dear Reviewer #1**. We thank the reviewer for this valuable comment. The term “New” refers to newly diagnosed TB patients, and we have now written it in full in the revised manuscript to ensure clarity for the reader.

**Reviewer’s comments:** Line 44: Add the words “in female TB patients” after the word “significantly”

**Authors’ response: Dear Reviewer #1**. We thank the reviewer for this helpful suggestion. We have added the words “in female TB patients” after the word “significantly” in line 44 to improve clarity.

**Reviewer’s comments:** Conclusion and recommendation: should be grounded in the findings of your study

**Authors’ response: Dear Reviewer #1**. We thank the reviewer for this important comment. We agree with the suggestion and have revised the conclusion and recommendation sections to ensure they are clearly grounded in the findings of our study.

**Reviewer’s comments:** Line 51 – 52: Change “Early diagnose” to “Early diagnosis” and state “why early diagnosis is crucial”

**Authors’ response: Dear Reviewer #1**. We thank the reviewer for this valuable comment. We have corrected the phrase to “early diagnosis” and clarified in the revised manuscript that early diagnosis of these hormonal changes is crucial in tuberculosis-positive patients to prevent complications such as impaired gonadal function related to hormonal disturbances.

**Introduction:**

**Reviewer’s comments:** Line 55: SPP name of the bacteria should be italicized

**Authors’ response: Dear Reviewer #1**. We thank the reviewer for the observation. The species name of the bacteria at line 55 has been italicized accordingly.

**Reviewer’s comments:** Line 56: Replace the first “it” with “TB”

**Authors’ response: Dear Reviewer #1**. We appreciate the reviewer’s suggestion. The first occurrence of “it” at line 56 has been replaced with “TB” to improve clarity.

**Reviewer’s comments:** Line 57: Change the word “damage” to “involve”, add the word “other” before the word “all”, replace “is” with the word “and”

**Authors’ response: Dear Reviewer #1**. We thank the reviewer for the helpful suggestions. At line 57, the word “damage” has been changed to “involve,” the word “other” has been added before “all,” and “is” has been replaced with “and” as recommended.

**Reviewer’s comments:** Line 61: Add the abbreviation COVID in brackets after the long form “(COVID)”

**Authors’ response: Dear Reviewer #1**. We appreciate the reviewer’s comment. At line 61, the abbreviation “COVID” has been added in brackets immediately after the long form, as suggested.

**Reviewer’s comments:** Line 65: The first sentence is not clear, rephrase

**Authors’ response: Dear Reviewer #1**. We thank the reviewer for pointing this out. The first sentence at line 65 has been rephrased for clarity.

**Reviewer’s comments:** Line 76: Delete the words “sex hormone” after “pathways”

**Authors’ response: Dear Reviewer #1**. We thank the reviewer for the observation. At line 76, the words “sex hormone” following “pathways” have been deleted as suggested.

**Reviewer’s comments:** Line 83: Put full stop after “(after puberty) and then start a new sentence as “The gender bias in………….”

**Authors’ response: Dear Reviewer #1**. We are grateful to the reviewer for the helpful suggestion. At line 83, a full stop has been inserted after “(after puberty),” and the subsequent text now begins with a new sentence: *“*The gender bias in *…”* as recommended.

**Reviewer’s comments:** Line 87: The first sentence of the paragraph lack connectivity; the author should re-write it clearly and put a reference.

**Authors’ response: Dear Reviewer #1**. We sincerely thank the reviewer for this valuable comment. The first sentence of the paragraph at line 87 has been rewritten to improve clarity and connectivity, and an appropriate reference has been added.

**Reviewer’s comments:** Line 101 – 108: The whole paragraph is not clear; the author should re-write it to be clear to the reader. Suggested: “Recently there has been a rise in global TB cases, deaths and spread of highly drug-resistant strains, providing alarming signals that other strategies will be needed to stop this endemic disease (29). Global control of TB can only be achieved through the concerted effort in the development of effective vaccines, improved diagnostics, as well as novel and shortened therapeutic regimens. The findings of this study will contribute to the identification of additional biomarkers that add up to the existing body of knowledge towards developing potential host markers for diagnostics, prognostics or vaccine development initiatives. Therefore, this study was aimed to assess sex hormone profile and associated factors in adult TB patients and compare with apparently healthy TB negative control.

**Authors’ response: Dear Reviewer #1**. We sincerely thank the reviewer for the insightful comment. The paragraph from lines 101–108 has been completely re-written for improved clarity and readability. The revised version now clearly contextualizes the study rationale and objectives as suggested.

**Materials and Methods:**

**Reviewer’s comments:** A lot of repetition have been observed these should be avoided.

**Authors’ response: Dear Reviewer #1**. We thank the reviewer for the observation. We have carefully reviewed the Materials and Methods section and removed repetitive statements to improve clarity and conciseness.

**Reviewer’s comments:** Line 112: add “selected” before “five”

**Authors’ response: Dear Reviewer #1**. We thank the reviewer for the suggestion. At line 112, the word “selected” has been added before “five” as recommended.

**Reviewer’s comments:** Line 114: Replace the words “used for data collection” with “involved in this study”

**Authors’ response: Dear Reviewer #1**. We thank the reviewer for the suggestion. At line 114, the phrase “used for data collection” has been replaced with “involved in this study” as recommended.

**Reviewer’s comments:** Line 117: Replace “by the year” with “as per year”

**Authors’ response: Dear Reviewer #1**. We thank the reviewer for the suggestion. At line 117, the phrase “by the year” has been replaced with “as per year” as recommended.

**Study population**

**Reviewer’s comments:** Line 121 – 123: The author should specify the study participants were matched by which characteristics

**Authors’ response: Dear Reviewer #1**. We thank the reviewer for this helpful comment. In the revised manuscript, we have clarified that study participants were matched by **age** to reduce potential confounding.

**Reviewer’s comments**: Line 138 – 152: Exclusion criteria: suggested to List or breakdown into slot (summarize)

**Authors’ response: Dear Reviewer #1**. We sincerely thank the reviewer for this constructive suggestion. To improve clarity, we have revised and summarized the exclusion criteria by presenting them in a list format for both TB-positive and TB-negative participants. This change makes the criteria easier to follow and avoids long, dense sentences.

**Reviewer’s comments**: Line 181 – 183: Advised to re-write the first and second sentences by combining the two in one sentence and state the kind of data collected using that questionnaire. Suggestion “The …….data were collected using a pre-structured Amharic translated questionnaire”

**Authors’ response: Dear Reviewer #1**. We sincerely thank the reviewer for this valuable suggestion. We have revised the text accordingly to combine the sentences and clearly state the type of data collected.

**Reviewer’s comments**: Line 184: Replace “selected” with “used as”

**Authors’ response: Dear Reviewer #1**. We thank the reviewer for this helpful observation. We have revised the text accordingly, and the word “selected” has been replaced with “used as.”

**Reviewer’s comments**: All the data that were collected in this study and their classification should be stated under the subheading “Data collection” and not under the subheading “operational definition”. This is for the reader to easily follow and understand. Eg how BMI was classified.

**Authors’ response: Dear Reviewer #1**. We sincerely thank the reviewer for this helpful suggestion. In the revised manuscript, we have moved all information regarding the collected data and their classification, including the classification of BMI, from the “Operational definition” section to a new “Data collection” subheading. This change improves clarity and allows readers to easily follow the study methodology.

**Reviewer’s comments**: The author need to state the tools used to measure weight, height and blood pressure (model, company and country of origin) eg. “Weight was measured with minimal clothing using a standard calibrated weighing scale (model, company, country of origin) Height was measured in the upright standing position using……………………….. and BMI was then calculated by the formula: weight in kg divided by height In meter squared”

**Authors’ response: Dear Reviewer #1**. We thank the reviewer for this helpful comment. As suggested, we have revised the Methods section to clearly state the instruments, model, company, and country of origin used for measuring weight, height, and blood pressure, along with a brief description of the procedures.

**Laboratory procedure and analysis**

**Reviewer’s comments**: Considering the circadian rhythm of sex hormones, the author need to state the time of the day blood samples for hormone assay were collected

**Authors’ response: Dear Reviewer #1**. We thank the reviewer for this important observation. In the revised manuscript, we have clarified the timing of blood sample collection to account for the circadian rhythm of sex hormones.

**Reviewer’s comments**: How were the blood/serum samples handled before the laboratory analysis of hormones?

**Authors’ response: Dear Reviewer #1**. We thank the reviewer for this important comment. Blood samples were collected following face-to-face interviews from participants after a minimum 8-hour fast. Five milliliters of venous blood were drawn from the medial cubital vein of the left arm using a 19-gauge syringe and transferred into gel-coated serum separator tubes labeled with unique ID numbers. Samples were collected between 8:00 and 10:00 AM to minimize circadian variation in hormone levels. The blood was allowed to clot at room temperature for 30 minutes, then centrifuged at 3000 RPM for 5 minutes to separate the serum. Serum samples were transported at 2–8°C to the University of Gondar Referral Comprehensive Specialized Hospital laboratory and analyzed on the same day using a Beckman Coulter DXI 800 automated clinical chemistry analyzer. These procedures ensured consistent pre-analytical handling and preservation of hormone integrity.

**Reviewer’s comments**: Line 211: replace “separated cells from” with “obtain”, Replace “The hormonal profile” with “The sex hormone profile”

**Authors’ response: Dear Reviewer #1**. We thank the reviewer for the helpful suggestions. We have revised the manuscript accordingly.

**Reviewer’s comments**: Line 212 – 214: Summarize the manufacturer in the bracket as “ (DXI800, Beckman Coulter inc, Danaher corporation company Brea, California United States)” to be placed after the word “analyzer” and then a full stop

**Authors’ response: Dear Reviewer #1**. We thank the reviewer for this helpful suggestion. We have revised the manuscript accordingly.

**Reviewer’s comments**: The author also need to specify the technique used for hormone assay.

**Authors’ response: Dear Reviewer #1**. We thank the reviewer for this important comment. In the revised manuscript, we have specified the technique used for hormone analysis. The sex hormone profile and cortisol were measured from serum using a **chemiluminescent immunoassay (CLIA) on the Beckman Coulter DXI 800 automated clinical chemistry analyzer (DXI800, Beckman Coulter Inc, Danaher Corporation, Brea, California, United States).**

**Statistical analysis**

**Reviewer’s comments**: Line 230: Add a reference for a statistical software used for data analysis

**Authors’ response: Dear Reviewer #1**. We sincerely thank the reviewer for this valuable suggestion. In line with PLOS ONE journal guidelines, the name, version, and manufacturer of the statistical software have been specified in the revised manuscript. However, we respectfully note that citing statistical software is generally not required, as software packages are considered widely available tools rather than primary sources. Therefore, we have provided complete details (software name, version, and manufacturer) without adding a reference citation, which we believe sufficiently informs readers and ensures reproducibility.

Operational definition
**Reviewer’s comments**: Summarize and most should put under data collection part following each specific data.

**Authors’ response: Dear Reviewer #1**. We sincerely thank the reviewer for this constructive suggestion. In the revised manuscript, we have summarized the operational definitions and moved the detailed measurement procedures under the Data Collection section, following each specific variable. This restructuring improves clarity, reduces repetition, and makes it easier for readers to follow the methodology.

**Results:**

**Socio demographic characteristics of study participants**

**Reviewer’s comments**: Line 285: change “aged” to “age”

**Authors’ response: Dear Reviewer #1**. We thank the reviewer for this careful observation. The term has been corrected from “aged” to “age” in the revised manuscript.

**Reviewer’s comments**: Line 285 – 286: You stated that there were a total of 300 participants (150 TB positive and 150 TB negative controls who were sex and age matched. Why again reported to have 162 (54%) males in this study. This is confusing, the author need to clarify

**Authors’ response: Dear Reviewer #1**. We thank the reviewer for this important comment. The sex- and age-matching was performed at the group level to ensure comparability between TB positive patients and TB-negative participants. The overall sex distribution (162 males and 138 females) is presented to provide descriptive statistics for the entire study population. To avoid confusion, we have revised the text for clarity. From male 162 (81 TB positive patients and 81 TB-negative participants), female 138 (69 TB positive patients and 69 TB-negative participants). That is why 150 were TB positive patients (81 male TB positive patients plus 69 female TB positive patients).

**Reviewer’s comments**: Line 285 – 2888: Suggested the author to directly start reporting the two groups and their characteristics i.e “ A total of 150 TB positive patients and 150 apparently healthy negative individual as comparison group matched by sex and age were included in this study. The mean age was 32.86 ± 12.88 and 32.81 ± 12.20 for TB positive patient and a comparison group respectively (Table 1)”.

**Authors’ response: Dear Reviewer #1**. We thank the reviewer for this helpful suggestion. In line with the comment, we have revised the section to directly report the study groups and their baseline characteristics.

**Clinical and behavioral characteristics of study participants**

**Reviewer’s comments**: Line 290 – 294: Edit as examples given above for clarity

**Authors’ response: Dear Reviewer #1**. We thank the reviewer for the suggestion. The section has been revised for clarity and consistency, and now directly reports the findings in line with the provided examples.

**Comparison of sex hormone profile among study participants**

**Reviewer’s comments**: Line 296 – 303: These 81 cases and 81 male control as well as 69 female cases and 69 female control should have been introduced early in the methodology part so that the reader can easily follow and understand not just appear over a sudden in the results section. This can be confusing to the reader, so the author is advised to revisit and do a needful amendments.

**Authors’ response: Dear Reviewer #1**. We thank the reviewer for this important observation. We agree that introducing the subgroup numbers (81 male TB positive patients with 81 TB- negative comparison group, and 69 female TB positive patients with 69 TB- negative comparison group) earlier in the methodology section provides better clarity and flow. Accordingly, we have revised the methodology to describe the male and female subgroup composition upfront. We have also refined the results section to directly present the findings with clear reference to these subgroups.

**Reviewer’s comments**: Better to indicate specific p –value for each hormone in the brackets i.e estradiol (p = 0.0…..) , LH (p=0.0..)

**Authors’ response: Dear Reviewer #1**. We thank the reviewer for this valuable suggestion. We have revised the results to indicate the specific p-values for each hormone, as recommended.

**Sex hormone profile in new TB cases, TB patients on treatment and controls**

**Reviewer’s comments**: Line 306 -323; The narration of results is not clear to the reader, the author advised to rephrase making simple and clear to the reader.

**Authors’ response: Dear Reviewer #1**. We sincerely thank the reviewer for this valuable comment. In response, we have carefully revised the results section (Lines 306–323) to simplify the narration and improve clarity for the reader. The revised text now presents the findings in a more structured manner, with clear separation of male and female results, concise reporting of post hoc comparisons, and direct linking of p-values to each hormone. We believe this revision enhances readability and makes the results easier to follow.

**Factors associated with alteration of sex hormone profile**

**Reviewer’s comments**: Line 325 – 326: This is supposed in the first place to be narrated in the data collection part.

**Authors’ response: Dear Reviewer #1**. We thank the reviewer for this helpful observation. As recommended, the information originally presented in Lines 325–326 has now been moved to the data collection section to ensure consistency and improve the overall flow of the manuscript.

**Reviewer’s comments**: Line 324 – 342: Advised to change the subheading to “Comparison of sex hormone according to treatment duration among new TB positive patients” and all the results narration under these lines should be within this same subheading

**Authors’ response: Dear Reviewer #1**. We thank the reviewer for this constructive suggestion. In line with the recommendation, we have revised the subheading in Lines 324–342 to read **“Comparison of sex hormones according to treatment duration among TB-positive patients.”** Furthermore, all results within this section have been reorganized under the revised subheading to ensure clarity and consistency.

**Reviewer’s comments**: The author is advised to be focused, concise and clear in results narration in the text within the results section

**Authors’ response: Dear Reviewer #1**. We sincerely appreciate the reviewer’s advice. In response, we have carefully revised the results section to make the narration more focused, concise, and clear. Redundant phrases have been removed, and the findings are now presented in a straightforward manner, with each result directly linked to the corresponding statistical test and p-value for ease of understanding.

**Reviewer’s comments**: The author should be consistent in their table formatting. Add units where appropriate in parentheses in each table: for example, Age (years) testosterone (ng/ml). Also need to write the data with equivalent number of decimal places for all tables (i.e one decimal place for all). Also the author need to amend the title of all tables by shortening it (reducing the number of words) e. g table 1 title can be written as “Socio-demographic characteristics of study participants (N = 300)”

**Authors’ response: Dear Reviewer #1**. We thank the reviewer for this valuable comment. In accordance with the recommendation, all tables have been revised to ensure consistency in formatting. Units of measurement have been added in parentheses where appropriate, and numerical data are now presented uniformly with one decimal place. Furthermore, table titles have been shortened to improve clarity and readability.

**Reviewer’s comments**: For Table 1. Amend Ages (years), add mean age on the first row, shorten the title

**Authors’ response: Dear Reviewer #1**. We thank the reviewer for the suggestion. The variable has been amended to “Age (years),” the mean age has been added in the first row, and the table title has been shortened as recommended.

**Reviewer’s comments**:  For Table 2. Amend cortisol (SI units), shorten title

**Authors’ response: Dear Reviewer #1**. We appreciate the reviewer’s comment. Cortisol has been amended to SI units, and the table title has been shortened as recommended.

**Reviewer’s comments**:  For table 3. Amend Testosterone (ng/ml), estradiol (SI unit),, P4 (SI unit),, LH (SI unit), FSH (SI unit), Shorten the title

For Table 4. Amend Testosterone (ng/ml), estradiol (SI unit),, P4 (SI unit),, LH (SI unit), FSH (SI unit), Shorten the title

For Table 5. Amend Testosterone (ng/ml), estradiol (SI unit),, P4 (SI unit),, LH (SI unit), FSH (SI unit), Shorten the title

**Authors’ response: Dear Reviewer #1**. We thank the reviewer for the constructive suggestions. For Tables 3, 4, and 5, the units for all hormones have been amended as requested. In addition, the titles of the tables have been shortened to ensure clarity and consistency with the journal’s formatting requirements.

**Reviewer’s comments**:  For Table 6, 7 and 8. Shorten the title

**Authors’ response: Dear Reviewer #1**. We thank the reviewer for this helpful comment. The titles of Tables 6, 7, and 8 have been revised and shortened to improve clarity and maintain consistency with the journal’s formatting requirements.

**Reviewer’s comments**:  Cross check all the tables for data and table title page overlap and correct.

**Authors’ response: Dear Reviewer #1**. We appreciate the reviewer’s observation. All tables have been carefully cross-checked to ensure data accuracy, consistency in units and decimal places, and alignment between table content and titles. Table titles have also been revised to avoid overlap with the main text, and redundant wording has been removed to improve clarity and readability.

**Reviewer’s comments**: The author need to put title for all the figures.

**Authors’ response: Dear Reviewer #1**. We thank the reviewer for the suggestion. In accordance with PLOS ONE’s formatting requirements, we have provided a separate, complete caption for each figure within the manuscript. Each caption serves to describe the figure content clearly.

**Discussion:**

**Reviewer’s comments**:  Line 386: The statement “in this age group” should be rephrased and state the specific age group being referred to. Here

**Authors’ response: Dear Reviewer #1**. We thank the reviewer for this valuable comment. We have revised the sentence to specify the age group. The updated text in the revised manuscript.

**Reviewer’s comments**: Line 386 – 387: Avoid repeating the objective and just directly start discussing your findings

**Authors’ response: Dear Reviewer #1**. We thank the reviewer for this constructive suggestion. We agree that restating the study objective was unnecessary. Accordingly, we have revised the text to remove the repetition and now begin the section by directly presenting the findings.

**Reviewer’s comments**: Line 393 – 395: The advised to continue using abbreviations i.e TB, FSH, LH

**Authors’ response: Dear Reviewer #1**. We thank the reviewer for this helpful suggestion. We have carefully revised the manuscript to ensure consistent use of the abbreviations “TB,” “FSH,” and “LH” throughout the text after their first mention.

**Reviewer’s comments**: Line 402: Suggested the sentence “However, opposing with the study…….” To be rephrased to “However this finding is contrary to the report of previous study in Ethiopia…………” and should be a continuation of the above paragraph.

**Authors’ response: Dear Reviewer #1**. We appreciate the reviewer’s constructive comment. The sentence at line 402 has been rephrased

**Reviewer’s comments**: Line 409: Add the words “and a” before the words “study conducted”

Line 410: Add the words “could be” after the word “This”

Line 413: Change the word “suggests” to “suggesting”

**Authors’ response: Dear Reviewer #1**. We thank the reviewer for the valuable suggestions. The recommended edits have been incorporated.

**Reviewer’s comments**: Line 417 - 422: This paragraph should be a continuation of the above paragraph and not a separate paragraph.

**Authors’ response: Dear Reviewer #1**. We thank the reviewer for the helpful suggestion. The paragraph in lines 417–422 has now been merged with the preceding paragraph, as recommended, to improve the logical flow and readability of the manuscript.

**Reviewer’s comments**: Line 419: Replace the words “methodological differences” with “differences in characteristics of study participants”

**Authors’ response: Dear Reviewer #1**. We thank the reviewer for the valuable suggestion. In line 419, the phrase “methodological differences” has been replaced with “differences in characteristics of study participants” as recommended.

**Reviewer’s comments**: Line 423 – 425: The sentence is not clear to the reader and need to be rephrased

**Authors’ response: Dear Reviewer #1**. We thank the reviewer for pointing out the lack of clarity in this sentence. The text in lines 423–425 has been revised.

**Reviewer’s comments**: Line 425 - 426"This difference may be attributed to the lifestyle difference" the paragraph reiterates. How might differences in lifestyle impact the relationship between TB and progesterone levels? Simply read and provide concrete evidence.

**Authors’ response: Dear Reviewer #1**. We thank the reviewer for this important comment. We apologize for the earlier error in extracting data from the Mexican study, which led to an inaccurate explanation. In our revision, we have corrected this section in the Discussion and now provide evidence-based clarification regarding the relationship between TB and progesterone levels, aligning our findings with those previously reported in the Mexican study.

**Reviewer’s comments**: Line 429 – 432: The sentence “Female TB patients…….” Is not clear, suggested to be rephrased as :” Female TB patients had statistically significant lower testosterone levels compared to controls. This is contrary to the finding of the previous study conducted in India (53)”. The difference could be due to difference in characteristics of study participants. The study subjects in the previous Indian study had higher prevalence of anemia as compared to participants in our study (75% vs 34% participants), and anemia negatively impacts testosterone level (54)”.

**Authors’ response: Dear Reviewer #1**. We thank the reviewer for this helpful suggestion. The sentence in lines 429–432 has been rephrased for clarity as recommended.

**Reviewer’s comments**: Line 432 – 436: The sentence “The difference that may be…….” be rephrased as “The difference could also be contributed by the inclusion of………….”

**Authors’ response: Dear Reviewer #1**. We appreciate the reviewer’s suggestion. The sentence in lines 432–436 has been rephrased as recommended.

**Reviewer’s comments**: Line 443 – 446: The paragraph should be a continuation of the above paragraph

**Authors’ response: Dear Reviewer #1**. We thank the reviewer for the suggestion. The paragraph in lines 443–446 has been merged with the preceding paragraph to improve the flow and continuity of the text.

**Reviewer’s comments**: Line 455 – 459: The paragraph should be a continuation of the above paragraph

**Authors’ response: Dear Reviewer #1**. We thank the reviewer for the suggestion. The paragraph in lines 455–459 has been merged with the preceding paragraph to improve flow and maintain continuity.

**Reviewer’s comments**: Line 460 – 471: The author advised to avoid too much repeat to report results in the discussion

**Authors’ response: Dear Reviewer #1**. We appreciate the reviewer’s feedback. The text in lines 460–471 has been revised to reduce repetition of results in the Discussion section, focusing instead on interpretation and comparison with previous studies.

**Reviewer’s comments**: Line 471 – 477: It is worthy to report and compare the prevalence between TB positive patients and TB negative comparison group and not the overall prevalence.

**Authors’ response: Dear Reviewer #1**. We thank the reviewer for this valuable comment. We agree that comparing the prevalence between TB-positive patients and the TB-negative comparison group provides more meaningful insights than reporting only the overall prevalence. Accordingly, we have revised the results section to present prevalence estimates separately for TB-positive and TB-negative participants. In addition, we have added comparative measures to highlight the differences between the two groups.

**Reviewer’s comments**: Line 513: Replacer the word “much” with “greater”

**Authors’ response: Dear Reviewer #1**. We thank the reviewer for this suggestion. We have replaced the word “much” with “greater” at line 513 as recommended.

**Reviewer’s comments**: The discussion sections should be generally revised so that the paper can be reconsidered

**Authors’ response: Dear Reviewer #1**. We thank the reviewer for the feedback. The Discussion section has been revised to reduce repetition of results, improve clarity, and focus on interpretation of findings in the context of existing literature. These changes enhance the manuscript’s readability and ensure that the discussion is directly linked to the study’s results.

**Strengths and limitation**

**Limitation of the study**

**Reviewer’s comments**: Line 521 – 522: The main aim of the study to determine sex hormone profile, how comes failure to measure hormones like leptin, thyroid stimulating hormone and thyroxine considered as a limitation of this study. The author need to clarify this.

**Authors’ response: Dear Reviewer #1**. We thank the reviewer for raising this point. While the primary aim of our study was to determine the sex hormone profile in TB patients, hormones such as leptin, thyroid-stimulating hormone (TSH), and thyroxine can influence metabolic and endocrine status, which in turn may affect sex hormone levels. Their measurement could have provided additional insights into potential confounding factors affecting the sex hormone profile.

Therefore, the absence of these measurements is acknowledged as a limitation, as it may restrict the ability to fully interpret all factors influencing hormonal changes in the study population.

**Reviewer’s comments**: Use of techniques other than liquid chromatography-tandem mass spectrometry (Gold standard) for sex for hormone assay in this study as well as measurement of total testosterone versus free testosterone could be other limitations to be addressed. The author need to verify these.

**Authors’ response: Dear Reviewer #1**. We thank the reviewer for the comment. In our study, sex hormones were measured using chemiluminescent immunoassay, and total testosterone levels were assessed in our study. We acknowledge that the use of techniques other than liquid chromatography–tandem mass spectrometry (the gold standard). This has been incorporated into the Limitations section of the manuscript.

**Conclusion and Recommendation**

**Reviewer’s comments**: Advised to re-write the conclusion and recommendation to be concise and clear to the reader, and both (conclusion and recommendation) should be emanating from the findings of this study

**Authors’ response: Dear Reviewer #1**. We thank the reviewer for the suggestion. The Conclusion and Recommendation sections have been revised to be concise, clear, and directly based on the study findings, emphasizing the key results and their implications for clinical practice and future research.

**Reviewer’s comments**: Line 542: Add the word “analysis” after the bracket

Line 545: Add the words “analysis tests” after the word “hormones”

**Authors’ response: Dear Reviewer #1**. We thank the reviewer for the valuable suggestions. The recommended edits have been incorporated.

**References**

**Reviewer’s comments**: References which require editing have been observed. These need to be edited, for example Reference number 3 and 25, have some words being abbreviated or it lack some important information.

**Authors’ response: Dear Reviewer #1**. We thank the reviewer for pointing this out. References requiring editing, including Reference numbers 3 and 25, have been carefully reviewed and corrected to ensure complete and accurate citation details according to the journal’s guidelines.

**Reviewer’s comments**: Also, several grammatical error have been observed throughout the document and require to be edited in the revision.

**Authors’ response: Dear Reviewer #1**. We appreciate the reviewer’s comment. The manuscript has been thoroughly proofread and edited to correct grammatical errors and improve overall clarity and readability.
